# Evolution and mutations predisposing to daptomycin resistance in vancomycin-resistant *Enterococcus faecium* ST736 strains

**DOI:** 10.1371/journal.pone.0209785

**Published:** 2018-12-21

**Authors:** Guiqing Wang, Fan Yu, Henry Lin, Karthikeyan Murugesan, Weihua Huang, Andrew G. Hoss, Abhay Dhand, Leslie Y. Lee, Jian Zhuge, Changhong Yin, Marisa Montecalvo, Nevenka Dimitrova, John T. Fallon

**Affiliations:** 1 Department of Pathology, New York Medical College, Valhalla, New York, United States of America; 2 Department of Pathology and Clinical Laboratories, Westchester Medical Center, Valhalla, Valhalla, New York, United States of America; 3 Philips Research North America, Cambridge, Massachusetts, United States of America; 4 Department of Medicine, New York Medical College, Valhalla, New York, United States of America; 5 Department of Pharmacy, Westchester Medical Center, Valhalla, New York, United States of America; LA BioMed at Harbor-UCLA, UNITED STATES

## Abstract

We recently identified a novel vancomycin-resistant *Enterococcus faecium* (VREfm) clone ST736 with reduced daptomycin susceptibility. The objectives of this study were to assess the population dynamics of local VREfm strains and genetic alterations predisposing to daptomycin resistance in VREfm ST736 strains. Multilocus sequence typing and single nucleotide variant data were derived from whole-genome sequencing of 250 *E*. *faecium* isolates from 1994–1995 (n = 43), 2009–2012 (n = 115) and 2013 (n = 92). A remarkable change was noticed in the clonality and antimicrobial resistance profiles of *E*. *faecium* strains between 1994–1995 and 2013. VREfm sequence type 17 (ST17), the prototype strain of clade A1, was the dominant clone (76.7%) recognized in 1994–1995. By contrast, clone ST736 accounted for 46.7% of VREfm isolates, followed by ST18 (26.1%) and ST412 (20.7%) in 2013. Bayesian evolutionary analysis suggested that clone ST736 emerged between 1996 and 2009. Co-mutations (*liaR*.W73C and *liaS*.T120A) of the *liaFSR* system were identified in all ST736 isolates (n = 111, 100%) examined. Thirty-eight (34.2%) ST736 isolates exhibited daptomycin-resistant phenotype, of which 13 isolates had mutations in both the *liaFSR* and cardiolipin synthase (*cls*) genes and showed high level of resistance with a daptomycin MIC_50_ of 32 μg/mL. The emergence of ST736 strains with mutations predisposing to daptomycin resistance and subsequent clonal spread among inpatients contributed to the observed high occurrence of daptomycin resistance in VREfm at our institution. The expanding geographic distribution of ST736 strains in other states and countries raises concerns about its global dissemination.

## Introduction

Vancomycin-resistant *Enterococcus faecium* (VREfm) belonging to the epidemic hospital clade A1, including strains of the clonal complex 17 (CC17) group, have emerged globally since the 1990’s and are now among the predominant group of enterococci causing nosocomial infections [1–3]. According to the US National Healthcare Safety Network (NHSN), enterococci are the second most common cause of nosocomial infections and 35.5% of hospital-associated enterococcal infections are vancomycin resistant [4]. Recent US hospitals surveillance studies show that approximately 80% of *E*. *faecium* clinical isolates during 2011 to 2014 were resistant to vancomycin (http://gis.cdc.gov/grasp/PSA/MapView.html). Moreover, infections with VREfm, compared to vancomycin-susceptible enterococci (VSE), are associated with increased morbidity, mortality, healthcare costs, and duration of hospital stay [5]. Therefore, VREfm has been listed by the US Centers for Disease Control and Prevention (CDC) as a target multidrug-resistant organism that requires improved infection control practice and infection reduction measures for both acute and long-term healthcare facilities [6]. Current antibiotic treatment of VREfm infections include linezolid, daptomycin, and tigecycline [7, 8]. Of these, daptomycin has potent bactericidal activity against enterococci, low risk of serious side-effects, and minimal drug-drug interactions. Daptomycin has been increasingly used in the US and other countries to treat serious staphylococcal and enterococcal infections, including infections caused by VREfm [1, 9–11]

Resistance to daptomycin is a serious clinical problem for treatment of severe VREfm infections, although it is still uncommon among clinical isolates worldwide [12, 13]. The mechanisms of daptomycin resistance in VREfm isolates remains to be fully elucidated. Daptomycin inserts into the plasma membrane in a calcium-dependent manner and subsequently disrupts the functional integrity of the cell membrane. Genomic sequencing analyses have revealed an association between daptomycin resistance and mutations in genes encoding the LiaFSR three-component regulatory cell envelope stress response pathway [14–20] and phospholipid biosynthesis enzymes cardiolipin synthase (Cls) in enterococci [14, 19, 21, 22]. It has been reported that mutations in the LiaFSR result in reduced binding of daptomycin to the cell surface in *E*. *faecium* [15, 17], and eventually lead to failures during daptomycin therapy with a subsequent mutation, most commonly in the *cls* gene [19, 20]. Among the mutations identified, substitutions in LiaR (W73C), LiaS (T120A) and Cls (H215R and R218Q) are among the most frequently observed [15, 20, 22], although mutations in either gene alone are not sufficient to confer a resistant phenotype in enterococci [16, 19, 21].

The VRE population in hospitals is highly diverse [1, 23, 24]. It is of interest for patient management and infection control to understand the antimicrobial resistance profiles, molecular epidemiology and transmission of local VRE populations in healthcare settings. However, current data on the clonal and temporal evolution of VRE clinical isolates in the US hospitals are limited. Westchester Medical Center (WMC) is a 652-bed tertiary-care medical center in the lower Hudson Valley of suburban New York City. VRE was first identified at WMC in May 1991 and an outbreak of VRE bloodstream infections in oncology patients was reported the same year [25]. Subsequent surveillance cultures for VREs in 1993–1995, 2009 and 2013 confirmed high rates of colonization (15–40%) among inpatients [26] (Wang *et al*., unpublished data). Recently, we identified a novel clone ST736 that accounts for 76.6% of daptomycin-nonsusceptible *E*. *faecium* isolates at our institution [27]. Since then, ST736 strains have been expanding to other hospitals in New York [28, 29], Washington [29, 30], Texas [15, 31], Maryland (https://pubmlst.org/efaecium/), Canada [32], countries in South America [15] and Caribbean [33], as well as Germany [34]. Moreover and the most worrisome, ST736 has been reported as the most common VREfm strains on hospital environmental surfaces and in laundry facility of some US teaching hospitals [31, 35]. The objectives of this study were to assess the evolution in clonality and antimicrobial susceptibility profiles of local VREfm populations, to explore the potential role of genomic mutations and nosocomial transmission in the emergence and spread of daptomycin-nonsusceptible ST736 strains.

## Materials and methods

### *Enterococcus faecium* clinical isolates

A total of 250 *E*. *faecium* clinical isolates, including 239 VREfm and 11 vancomycin-susceptible *E*. *faecium* (VSEfm) isolates, were included in this study. All isolates were recovered from patients with an infection, with the exception of one from an environmental sample, in a tertiary-care medical center of suburban New York City. Isolates were collected from three different study periods: 1) 1994–1995 (n = 43): Forty-nine VRE isolates were randomly selected from a collection of saved isolates during an outbreak investigation from July 1994 through July 1995. Of these, 43 *E*. *faecium-*VRE isolates were included, while 6 *E*. *faecalis*-VRE isolates were excluded in the analysis; 2) 2013 (n = 92): this included all non-duplicate, consecutive VREfm isolates recovered from January through October 2013; and 3) 2009–2012 (n = 115): which comprised all confirmed daptomycin-nonsusceptible *E*. *faecium* (DNSEfm) isolates and representing daptomycin-susceptible isolates spanning different months of each year. One *E*. *faecium* isolate per patient was chosen unless there were two isolates from the same patient with different sequence types (ST) or with different susceptibility (susceptible vs. nonsusceptible) to daptomycin. All enterococci isolates were identified by conventional biochemical tests and confirmed using the MALDI Biotyper CA system (Bruker, Billerica, MA) and/or DNA sequencing analysis of 16S rRNA gene. Antimicrobial susceptibilities of *E*. *faecium* isolates were measured by broth microdilution with the MicroScan 96*Plus* test system and daptomycin-nonsusceptibility (MIC > 4 μg/mL) was confirmed by E-test as described previously [27].

### Next-generation sequencing (NGS) and data analysis

DNA extraction, quantitation and library preparation were carried out as described previously [27]. Paired-end sequencing was performed using either Illumina MiSeq (2×250 bp) at New York Medical College (Valhalla, NY) or HiSeq 2000 (2×100 bp) at the Cold Spring Harbor Laboratory (Cold Spring Harbor, NY). Illumina raw sequencing reads were trimmed to clip adapters and low-quality bases (Phred score <10) using Trimmomatic v0.36 [36]. The trimmed reads were examined for both sample swapping and taxonomic abundance using Kraken v0.10.5-beta [37] and assembled *de novo* with MEGAHIT v1.1.2 [38]. From the assemblies, the multilocus sequence typing (MLST)-based clonality was determined *in silico* using BLAST-based tool (https://github.com/tseemann/mlst) and the PubMLST database (https://pubmlst.org/) [39]. Sanger DNA sequencing was used for allele confirmation in some isolates when NGS failed to retrieve MLST or new alleles were found. Trimmed sequencing reads were also aligned to the reference genome of a local *E*. *faecium* ST736 strain E39 (RefSeq accession number NZ_CP011281.1) to produce a reference-based whole-genome alignment including single nucleotide variant (SNV) and indels (insertions and/or deletions) with Snippy v3.1 (https://github.com/tseemann/snippy).

### Bayesian phylogenetic analyses

To investigate the phylogenetic relationship and the divergence time of *E*. *faecium* circulating at local patient population, Bayesian Evolutionary Analysis by Sampling Trees 2 (BEAST 2) v2.4.7 [40] was used to jointly estimate molecular clock phylogeny, rate of evolution, divergence times and other evolutionary parameters. By using Snippy, a reference-based whole-genome alignment was generated to include all 250 *E*. *faecium* samples. To reduce excessive computational load, three isolates with low depth of genomic coverage were excluded, which resulted a final of 247 samples and 487,932 sites (479,196 invariant sites) for the BEAST analysis.

Bayesian Markov Chain Monte Carlo (MCMC) analysis was performed using the Hasegawa-Kishino-Yano (HKY) [41] nucleotide substitution model, along with a coalescent constant population tree prior and a discrete gamma-distributed among-site-rate-variation model with four categories [42]. A strict molecular clock model was employed, and tip dates were set from the sample collection dates. The MCMC chain was run for 1.5 billion states and sampled every 100,000 states. 10% sampled states were discarded as burn-in. Convergence was assessed using Tracer v1.7 [43], and effective sample size (ESS) values above 200 were accepted. A maximum clade credibility (MCC) tree was summarized with TreeAnnotator (available in the BEAST 2 v2.4.7 package) and visualized in Figtree v1.4.4 (http://tree.bio.ed.ac.uk/software/figtree/).

### Mutation analysis of *liaFSR* and *cls* genes

The assembled genome of each isolate with reference to the *E*. *faecium* strain E39 (ST736, accession numbers CP011281-CP011285, CP015123) was blasted against the *liaS* (1,069 bp) and the *liaR* (634 bp) gene sequences of strain DO (ST18, accession no. CP003583), and the *cls* (1,452 bp) gene sequence of strain UW7606x64/3 TC1 (ST192, accession no. CP013009). Corresponding gene sequences for each isolate were extracted. SNPs of *liaFSR* and *cls* genes in all *E*. *faecium* isolates and genetic alterations between isolates of each isogenic pair were called out using SNP-sites (http://dx.doi.org/10.1099/mgen.0.000056), in association with daptomycin resistance from the CARD database (https://card.mcmaster.ca/).

### Statistical analysis

The Fisher’s exact test from the GraphPad Prism software (version 7.0) was used to determine the statistical significance of association between the different STs of *E*. *faecium* with distinct clinical and microbiological characteristics.

### Accession numbers

The complete genomes of four representative ST736 strains (E39, E232, E243, and E240) were deposited to GenBank with accession numbers of CP011281-CP011285, CP015123, and CP017787 to CP017801. The untrimmed Illumina raw sequencing reads for all 250 *E*. *faecium* isolates were uploaded to GenBank under BioProject PRJNA386994.

## Results

### Clonality of VREfm population from 1994–1995 vs. 2013

To determine the population dynamics and evolution of VREfm clinical isolates and correlation between distinct clones and daptomycin susceptibility, we determined the sequence types (ST) of 92 non-duplicated, consecutive VREfm isolates from 86 inpatients hospitalized during January through October 2013. Two of these patients carried VREfm isolates with different STs (ST412 and ST736), while four patients had VREfm isolates with one isolate susceptible and another nonsusceptible to daptomycin. Eight distinct STs with three dominant clones (ST736, ST18 and ST412) were recognized among the 2013 VREfm clinical isolates. Of these, 43 (46.7%) isolates belonged to ST736, followed by ST18 (n = 24, 26.1%), ST412 (n = 19, 20.7%) and five other STs each with one to two isolates (Table A in S1 File).

**Fig 1:**
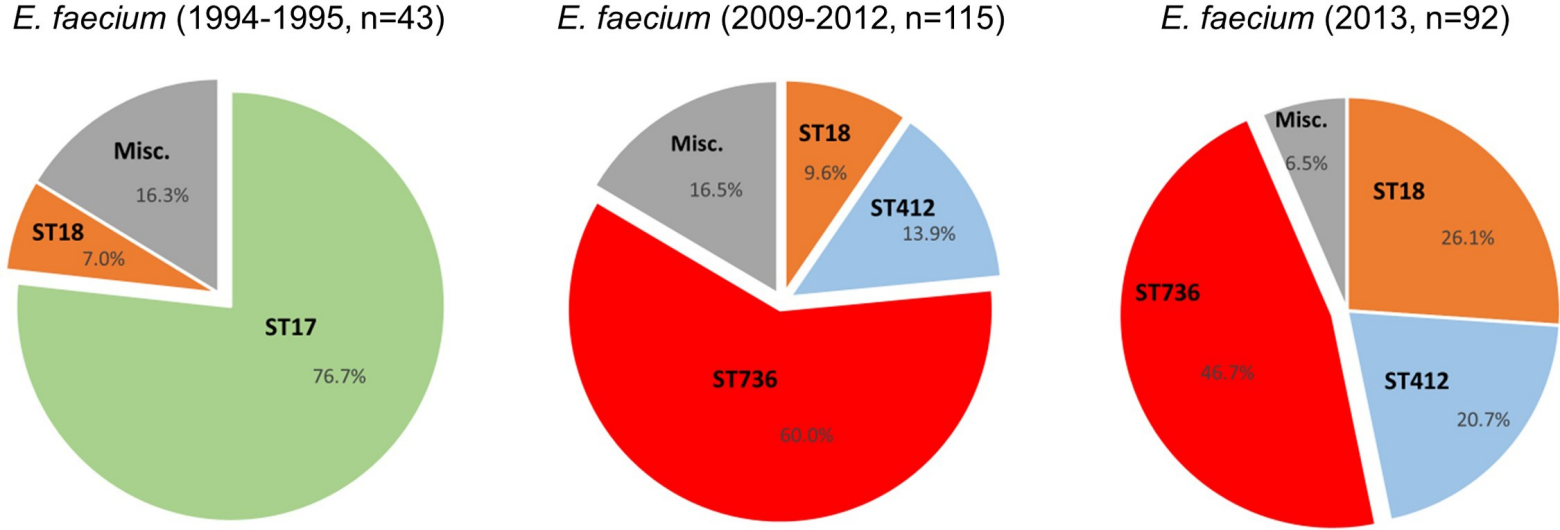
Clonality of VREfm from 1994–1995, 2009–2012 and 2013. The sequence types (STs) of VREfm were derived from whole-genome sequences as described in the text. Refer to Table A in S1 File for more information on clonal distribution of *E*. *faecium* clinical isolates (1994–2013).

For comparison, we also analyzed 43 VREfm isolates randomly selected from patients in 1994–1995, the earliest collection of VREfm isolates available for this study. ST736 was not detected in these VREfm isolates. By contrast, 33 of 43 (76.7%) VREfm isolates from 1994–1995 were ST17, a prototype of *E*. *faecium* clonal complex 17 (CC17) or clade A1 (**Fig 1**). Additional clones detected among the 1994–1995 VREfm isolates included ST18 (n = 3, 7.0%), ST16 (n = 2, 4.7%) ST535 (n = 2, 4.7%), and three other STs (ST20, ST186 and ST280) with one isolate each.

In addition, the clonality of 115 *E*. *faecium* isolates from 2009 to 2012 were examined. ST736 strains were detected in all years between 2009 and 2012. The number of *E*. *faecium* isolates from different study years and distribution in clonality are summarized in Table A in S1 File.

### Antimicrobial susceptibility profiles of VREfm from 1994–1995 vs. 2013

The antimicrobial susceptibility profiles of VREfm isolates from 1994–1995 and 2013 are shown in **Table 1**. A significant change in antimicrobial resistance profiles was observed between VREfm isolates from 1994–1995 and from 2013. All 43 VREfm isolates from 1994–1995 were susceptible to linezolid and daptomycin. By contrast, one of 92 (1.1%) VREfm isolates from 2013 were resistant to linezolid (*p* > 0.05) and 26 (28.3%) isolates from 2013, including 20 ST736 and 6 non-ST736 strains, were resistant to daptomycin (*p* < 0.0001). A point mutation G2576T of the 23S rRNA [44] was confirmed in the linezolid-resistant VREfm isolate (E243) from 2013. In addition, the 2013 VREfm isolates showed higher resistance rate to tetracycline than those from 1994–1995 (90.2% vs. 32.6%, *p* < 0.0001). Of 14 tetracycline-resistant VREfm isolates from 1994–1995, 13 isolates carried *tet(M)* resistance gene and one isolate possessed *tet(L)* gene. The number of VREfm isolates carrying both *tet(M)* and *tet(L)* was increased significantly from 14.3% (2 of 14) in 1994–1995 to 43.4% (36 of 83) in 2013 (*p* < 0.01). All VREfm isolates from 1994–1995 and 2013 carried the *vanA* gene with an exception of one ST186 isolate (E508) from 1995, in which a *vanB* gene was detected.

**Table 1:** Antimicrobial resistance profiles of vancomycin-resistant *E*. *faecium* (VREfm) clinical isolates, 1994–1995 (n = 43) versus 2013 (n = 92)^a^.

Antimicrobial agent	1994–1995 VREfm (No., %)	2013 VREfm (No., %)	*p* value
**Ampicillin**	41 (95.3)	92 (100)	0.0998
**Daptomycin**	0	26 (28.3)	<0.0001
**Linezolid**	0	1 (1.1)	1.0000
**Tetracycline**	14 (32.6)	83 (90.2)	<0.0001
**Vancomycin**	43 (100)	92 (100)	1.0000

^a^ All VREfm isolates from 1994–1995 and 2013 were resistant to erythromycin and levofloxacin. The data shown in the parenthesis are percent of isolates with resistance to specific antibiotics.

### Evolutional analysis on the emergence of VREfm ST736 clone

**Fig 2:**
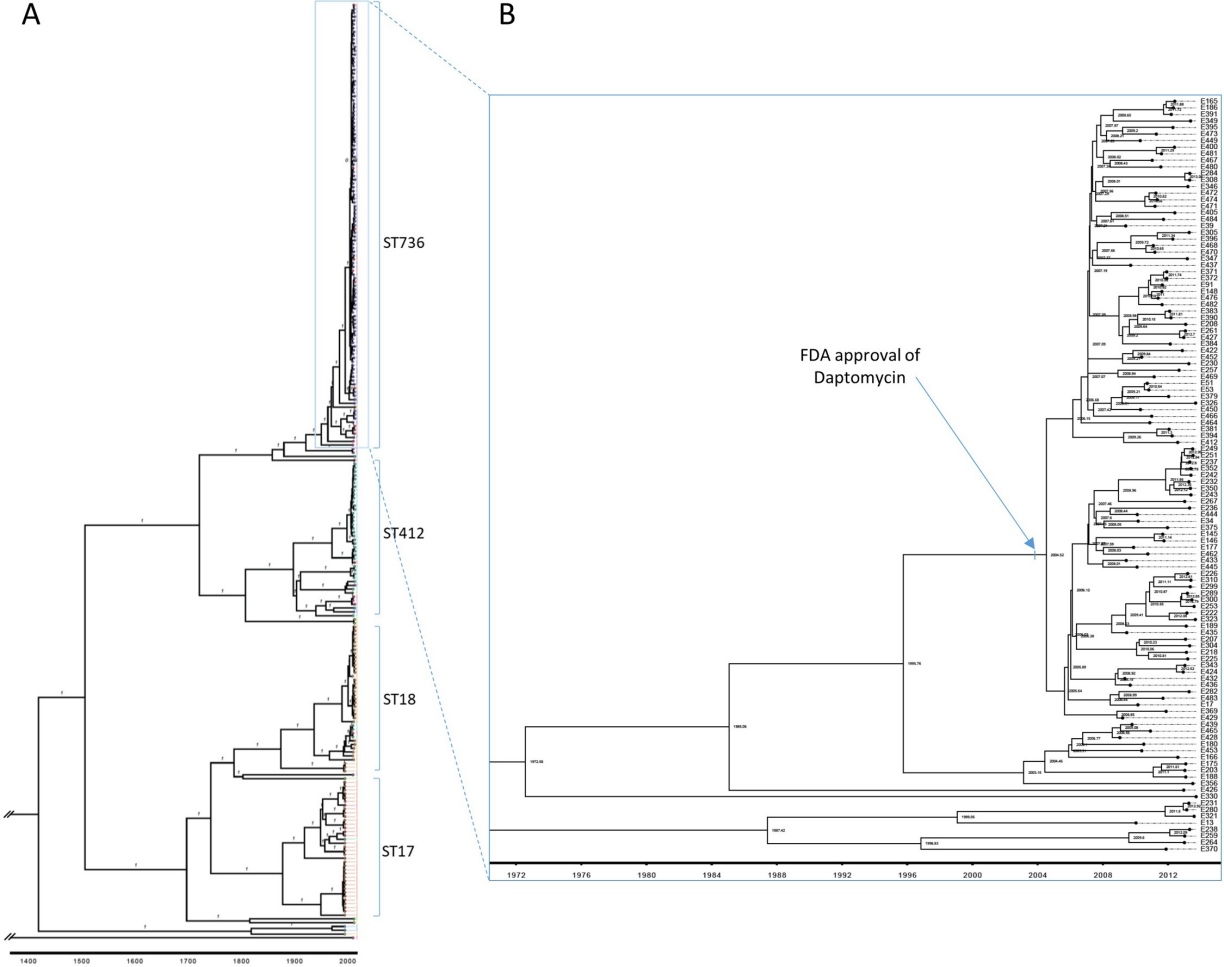
Evolutional analysis on the emergence of VREfm ST736 strains. (A) Maximum clade credibility (MCC) tree inferred using BEAST 2 with a whole-genome-based alignment that includes 247 *E*. *faecium* isolates and 487,932 sites. Bayesian analysis was run using a strict molecular clock model and with an HKY+G nucleotide substitution model assuming a coalescent constant population tree prior. Numbers above branches indicate posterior probabilities. (B) A zoomed-in version of the whole MCC tree showing the ST736 clade and the estimated divergence time of each node in year.

To explore the evolution of VREfm and estimate the possible emergence time of VREfm ST736 at our institution, we selected *E*. *faecium* isolates with a minimum breadth coverage of >60% reference genome, and performed the Bayesian evolutionary analysis. The final analysis included 487,932 sites (479,196 invariant sites) from the whole-genome alignment of 247 *E*. *faecium* isolates. As shown in **Fig 2**, BEAST analysis suggested that VREfm ST736 strains might have emerged between 1996 and 2009, most likely between 2004 and 2006 at our institution based on BEAST analysis. In addition, multiple subclusters were observed in the major branch of ST736 groups, each with one or more closely related isolates.

### Association between distinct VREfm clones and daptomycin susceptibility

**Fig 3:**
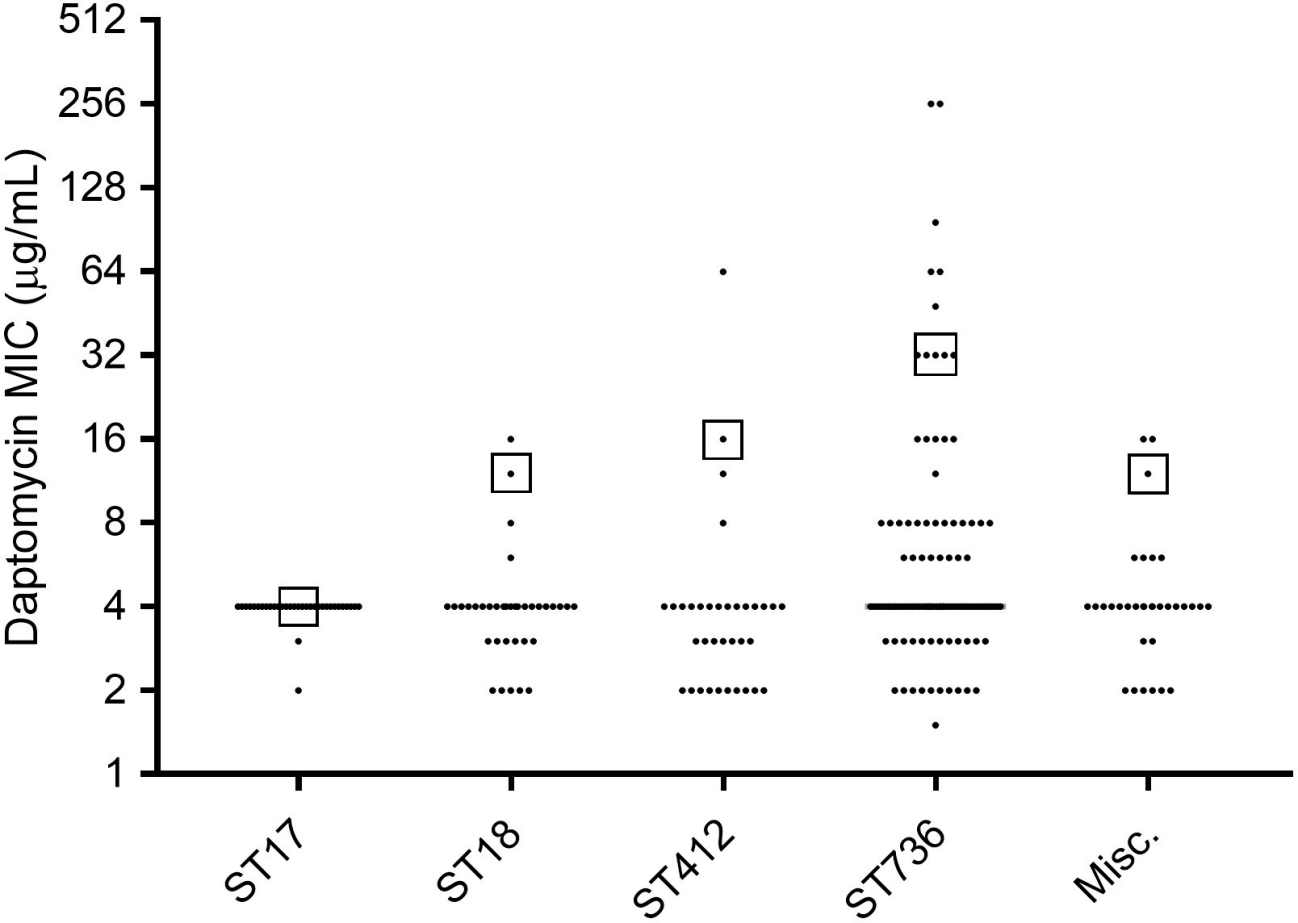
Distribution of daptomycin MIC among different sequence type (ST) of *E*. *faecium* strains from 1994 to 2013. The cumulated numbers of *E*. *faecium* strains included in this analysis were ST17 (n = 3), ST18 (n = 38), ST412 (n = 35), ST736 (n = 111) and miscellaneous (n = 33). The square box symbol represents the daptomycin MIC_90_ of each ST strains. Refer to Table C in S1 File for distribution of daptomycin MIC of major *E*. *faecium* clones for each time period of evaluation.

To further our understanding [27] on the association between different VREfm clones and daptomycin nonsusceptibility, we analyzed 92 VREfm isolates representing an unbiased collection of all hospitalized patients with VREfm infections from January through October 2013. Interestingly, clone ST736 strains accounted for only 46.7% (43 of 92) of all VREfm isolates examined in 2013 but 76.9% (20 of 26) of DNSEfm from this study period (*p* = 0.0002). The prevalence of ST736 as DNSEfm (46.5%, 20 of 43) was significantly higher than that of non-ST736 VREfm isolates from the same study period (6 of 49, 12.2%, *p* < 0.001). The distribution of daptomycin MICs of different *E*. *faecium* clones were shown in **Fig 3** and Tables B and C in S1 File. The daptomycin MIC_90_ of ST736 strains was 32 μg/mL, which was significantly higher than those from other non-ST736 strains (MIC_90_: 8 μg/mL, *p* <0.01).

### Mutations in *liaFSR* and *cls* and daptomycin resistance

The mutations and frequency of *liaFSR* and *cls* reported to be associated with daptomycin resistance in *E*. *faecium* [45] were analyzed and compared between ST736 and non-ST736 isolates (**Table 2**). Two mutations in *liaFSR* (W73C and T120A) and several mutations in *cls* (N13I, N13T, A20D, H215R, R218Q) were detected, while the remaining mutations described by Arias *et al*. [14] and listed in the CARD database were not seen in our isolates. Strikingly, all ST736 isolates (n = 111, 100%) examined had co-mutations (W73C and T120A) in the *liaFSR*, which was significantly higher than that detected in non-ST736 isolates (17 of 139, 12.2%, *p* < 0.0001).

**Table 2:** Frequency of the *liaRS* and *cls* mutations among daptomycin-nonsusceptible and daptomycin-susceptible *E*. *faecium* clinical isolates^a^.

			*liaRS* mutations			*cls* mutations		
*E*. *faecium*	ST group	No. of isolates	No.	%	*p* value^b^	No.	%	*p* value
**DNSEfm**	ST736	38	38	100.0	<0.0001	13	34.2	0.5076
	Non-ST736	15	4	26.7		3	20.0	
	All STs	53	45	84.9	Footnote^c^	16	30.2	Footnote^d^
**DSEfm**	ST736	73	73	100.0	<0.0001	0	0	>0.05
	Non-ST736	124	13	10.5		1	0.8	
	All STs	205	107	52.2		1	0.5	
**Total**	ST736	111	111	100.0	<0.0001	13	11.7	0.0041
	Non-ST736	139	17	12.2		4	2.9	
** **	All STs	250	128	51.2		17	6.8	

^a^ DNSEfm: Daptomycin-nonsusceptible *E*. *faecium*; DSEfm: Daptomycin-susceptible *E*. *faecium*

^b^ The *p* value between ST736 and non-ST736 isolates.

^c^ *p* < 0.001 between DNSEfm (84.9%) and DSEfm (52.2%) isolates.

^d^ *p* < 0.001 between DNSEfm (30.2%) and DSEfm (0.5%) isolates.

The overall prevalence of mutations (N13I, N13S, N13T, A20D, H215R, R218Q) in *cls* was 6.8% (17 of 250), which was much higher in ST736 (11.7%, 13 of 111) than in non-ST736 isolates (2.9%, 4 of 139, *p* = 0.0041, Table 2). Significantly higher prevalence of mutation in *cls* was also noticed in DNSEfm (30.2%, 16 of 53) than in DSEfm isolates (0.5%, 1 of 197, *p* <0.0001).

Thirty-eight of 111 (34.2%) ST736 isolates with *liaFSR* mutations were resistant to daptomycin, while all 13 ST736 isolates with both *liaFSR* and *cls* mutations exhibited a daptomycin-resistant phenotype. Moreover, ST736 isolates with *cls* mutations (n = 13) appeared to confer high resistance to daptomycin (MIC_50_ of 32 μg/mL), compared to those without *cls* mutations (MIC_50_: 8 μg/mL) (**Table 3**and Table B in S1 File).

**Table 3:** Common mutations and frequency of the *liaFSR* and cardiolipin synthase (*cls)* genes detected among daptomycin-nonsusceptible VREfm clinical isolates.

*E*. *faecium* clone(s)	No. of isolates	Gene(s)	Mutation	No. of isolates with mutation (%)	Daptomycin MIC range (μg/mL)
**ST736**	38	*liaFSR*	liaR.W73C	38 (100)	6 - >256
		*liaFSR*	liaS.T120A	38 (100)	6 - >256
		*cls*	N13I (A38T)	1 (2.6)	32
		*cls*	N13S (A38G)	1 (2.6)	32
		*cls*	N13T (A38C)	6 (15.8%)	16–96
		*cls*	A20D	1 (2.6)	>256
		*cls*	H215R	3 (7.9)	8–16
		*cls*	R218Q	1 (2.6)	8
					
**Non-ST736**	15	*liaFSR*	liaR.W73C	4 (26.7)	6–16
		*liaFSR*	liaS.T120A	4 (26.7)	6–16
		*cls*	N13S	1 (6.7)	16
		*cls*	H215R	1 (6.7)	12
** **		*cls*	R218Q	1 (6.7)	16

Thirty of 111 (27.0%) VREfm ST736 isolates had prior exposure to daptomycin within 12 months before their recovery from patients. VREfm ST736 isolates from patients with prior daptomycin exposure were more likely to be resistant to daptomycin (20/30, 66.7%) than those without prior exposure (18 of 81, 22.2%, p < 0.0001) with a relative risk of 3.0 (95% confident interval: 1.85–4.84). Moreover, 10 of 13 ST736 isolates with *cls* mutations had prior use of daptomycin. As shown in **Table 4**, four of six patients with isogenic pair of ST736 isolates developed resistance during daptomycin therapy by inducing *cls* mutations.

**Table 4:** Daptomycin exposure and development of resistance during therapy in patients with isogenic pairs of ST736 strains.

Year	Patient	ST736 isolate	Source	Interval between isolation (days)	Prior daptomycin exposure	Daptomycin MIC (μg/mL)	*liaFSR* mutation(s)	*cls* mutation(s)
**2010**	107	E51	Blood		-	4	liaR.W73C, liaS.T120A	-
		E53	Wound	36	+	64	liaR.W73C, liaS.T120A	N13I
**2011**	74	E148	Blood		+	2	liaR.W73C, liaS.T120A	
** **		E91	Blood	9	+	64	liaR.W73C, liaS.T120A	N13I
** **	124	E145	Blood		+	2	liaR.W73C, liaS.T120A	
** **		E146	Urine	26	+	32	liaR.W73C, liaS.T120A	N13I
**2013**	161	E232	Pelvic		-	4	liaR.W73C, liaS.T120A	
** **		E243	Peritoneal fluid	44	+	>256	liaR.W73C, liaS.T120A	
** **	184	E352	Wound		-	2	liaR.W73C, liaS.T120A	
** **		E242	Wound	4	+	8	liaR.W73C, liaS.T120A	
** **	185	E300	Wound		-	3	liaR.W73C, liaS.T120A	
		E253	Wound	47	+	>256	liaR.W73C, liaS.T120A	A20D

Two VREfm ST736 isolates (E243 and E253) had a daptomycin MIC of >256 μg/mL. Comparative genomic analysis of isogenic pair revealed no mutations in the chromosome of E243. For isolate E253, mutations in the *cls* (A20D) and a tyrosine kinase (P90H) as well as frameshift in a gene coding the TlyA family rRNA (cytidine-2'-O)-methyltransferase were also identified on the chromosome (**Table 5**). In addition, mutations in different insertion sequence (IS) family transposases were noticed in the plasmids of both isolates.

**Table 5:** Genetic alterations of VREfm isolates with high daptomycin MIC as compared to the corresponding isogenic pair.

Isolate	Daptomycin MIC (μg/mL)	GenBank accession no.	Genome	Position^a^	Locus tag (E39)	Mutation/amino acid change	Predicted function
**E243**	>256	NZ_CP011281	Chromosome			No mutations detected	
		NZ_CP011282	Plasmid-1	136763	XM37_RS14230	c.758_759delTGinsCC p.Leu253Ser	IS982 family transposase
**E253**	>256	NZ_CP011281	Chromosome	792300	XM37_RS03700	c.698dupG p.Glu234fs	TlyA family rRNA (cytidine-2'-O)-methyltransferase
				1033952	XM37_RS05010	c.59C>A p.Ala20Asp	Cardiolipin synthase
				1842891	XM37_RS08900	c.269C>A p.Pro90His	Tyrosine kinase
		NZ_CP011282	Plasmid-1	17787	XM37_RS13585	c.819A>C p.Glu273Asp	IS30 family transposase
				79592	XM37_RS13930	c.116G>A p.Gly39Asp	IS6 family transposase IS1216E
				93719	XM37_RS14010	c.67C>T p.Pro23Ser	Transposase
				147157	XM37_RS14285	c.56A>G p.Asp19Gly	IS6 family transposase

^a^ Position numbers corresponded to the nucleotide no. of isolate E39.

### Nosocomial transmission of VREfm ST736

**Fig 4:**
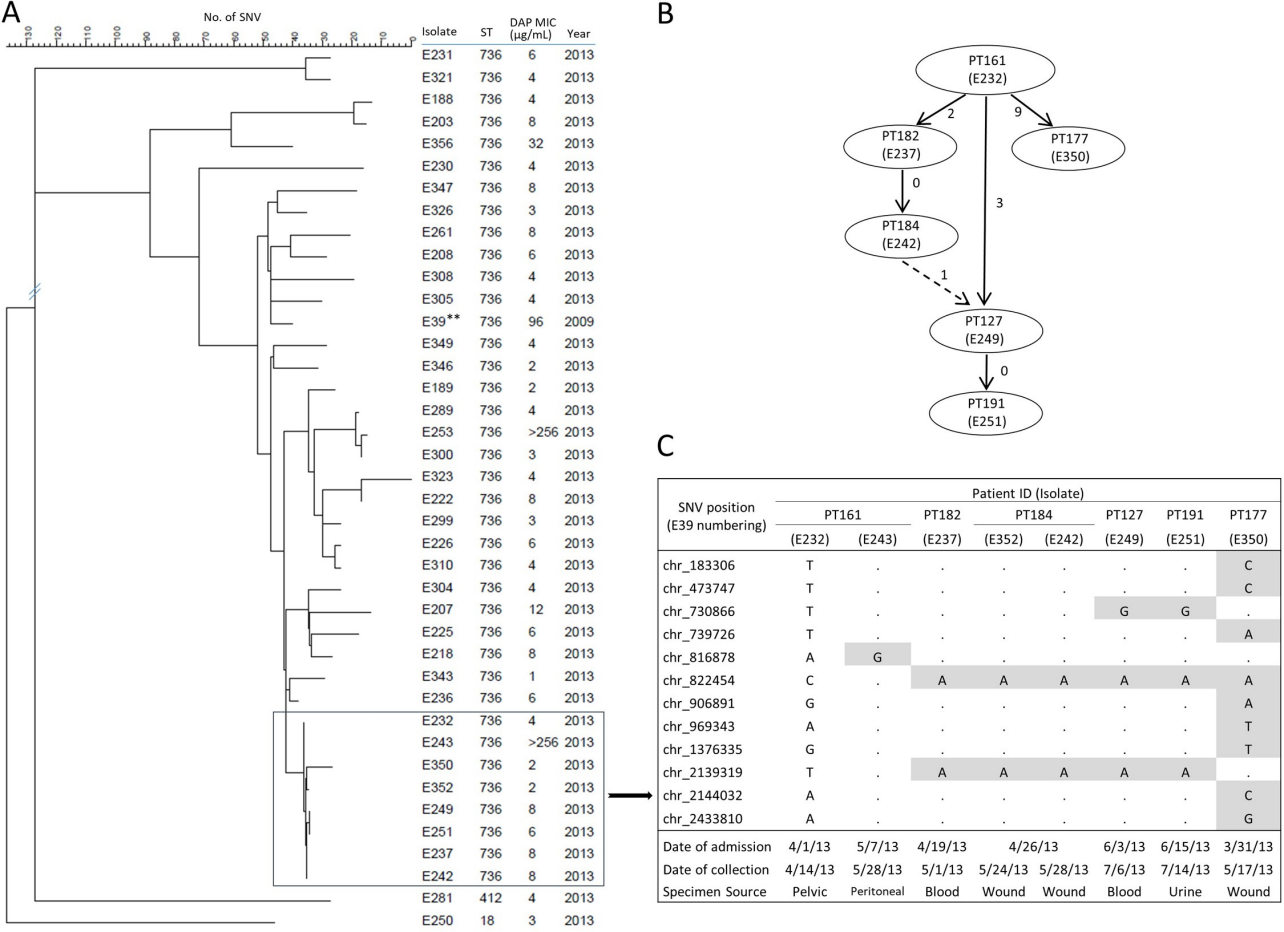
Possible nosocomial transmission of ST736 VREfm isolates among inpatients. (**A**). Chromosomal SNVs-based phylogenetic tree of representing VREfm ST736 isolates from 2013 (n = 37). Tree was constructed using the neighbor-joining method. (*): VREfm E39 was used as reference isolate. The two non-ST736 isolates were used as out of group control. (**B**) Possible transmission route for a selected group of 6 patients with 8 closely related *E*. *faecium* isolates. Solid arrow: transmission with epidemiological evidence [i.e., stayed in the emergency room (PT161 and PT182), on the same bed (PT161 and PT127), in the same medical intensive care unit (PT182 and PT184), or hospitalized during the same time period with overlap for at least 2 weeks (PT127 and PT191, PT161 and PT177)]; dashed arrow: patients had no direct epidemiological links. All transmissions with the exception between patients PT161 and 127 were suggested by SNVs-based minimum spanning tree [46]; Next to each arrow between two samples is the number of SNV differences between the samples. (**C**) SNVs identified based on whole-genome sequencing with clinical data of the patients. Sequences identical to those from isolate E232 were shown in dot (.); SNVs were listed and shadowed in gray.

To assess potential nosocomial transmission in spreading DNSEfm, a phylogenetic tree with ST736 isolates from 2013 was constructed (**Fig 4A**). Notably, 31 of 37 ST736 VREfm isolates analyzed fell into a closely related cluster with less than 50 SNVs among different isolates. Of these, 8 VREfm isolates from 6 patients revealed a distinct subcluster with less than 10 SNVs (0–9 SNVs). As shown in **Fig 4**, both epidemiological links (e.g., patients staying in the same hospital bed/room, visiting the same clinic on the same day, or hospitalized during the same period with at least two weeks of overlap) and SNVs-based genomic evidence were identified among the 6 patients in this subcluster, supporting the occurrence of nosocomial transmission [46].

## Discussion

In this study, we sought to determine if ST736 had long been in existence or only recently emerged at our institution. Comparative genomic analysis of clinical isolates revealed a dramatic change in the clonality and antimicrobial resistance profiles of local *E*. *faecium* population from 1994–1995 to 2013. The dominant clone of VREfm isolates in 1994–1995 was ST17 (77%), a prototype strain of CC17 or clade A1, when VREfm started emerging in the mid-1990 in the US [47]. No ST736 clones were identified among VREfm isolates from 1994–1995. By contrast, ST736 strains became predominant, accounting for 46.7% of local VREfm isolates in 2013. *E*. *faecium* ST18 appeared to be only clone continuously detectable in both study periods (1994–1995 and 2013). VREfm ST736 continues to be the dominant clone among isolates of 2014 (37.7%) and 2015 (36.9%) examined (data not shown). Bayesian evolutionary analysis, with additional *E*. *faecium* isolates from 2009 to 2012, provided evidence for the recent emergence of VREfm clone ST736 between 1996 and 2009. Based on the BEAST analysis, ST736 strains most likely emerged between 2004 and 2006. Due to the lack of VREfm isolates saved between 1996 and 2008, it would be difficult to determine the precise year of ST736 emergence.

In this population-based study, non-duplicate VREfm isolates from all patients admitted during January to October 2013 were analyzed. This eliminated potential bias in selecting isolates. In 2013, ST736 strains accounted for only 46.7% (43/92) of all VREfm but 76.9% (20/26) of daptomycin-resistant VREfm isolates. This confirms that the observed high occurrence of DNSEfm in clone ST736 strains is not simply due to its high prevalence at our institution [27]. In 2013, 20 of 43 (46.5%) ST736 isolates were DNSEfm, while only 6 of 49 (12.2%) non-ST736 isolates were DNSEfm (*p* <0.0001). The probability of an ST736 isolate to be daptomycin-nonsusceptible is significantly higher than that of a non-ST736 isolate (*p* = 0.0002) with an estimated relative risk of 3.9 (95% confidence interval: 1.7 to 8.9). Our data highlight the risk of ST736 clone in dissemination of daptomycin resistance and challenge in management of patients infected with ST736 VREfm strains.

The mechanisms of resistance to daptomycin appear to be diverse for VREfm strains with different genetic backgrounds [10, 45, 48]. Both *de novo* and developed resistance during daptomycin therapy have been reported [10]. Among several genomic pathways proposed, a hierarchical adaption via sequential mutations in the *liaFSR* signaling system and *cls* appears to be the most common mechanism associated with daptomycin resistance identified in enterococci [15, 16, 19, 49–51]. Since most of published data are based on analysis of a limited number of clinical isolates and/or laboratory-derived mutants, our current report represents the largest collection of *E*. *faecium* clinical isolates that can serve as an independent validation on the correlation between some previously described mutations and daptomycin resistance. Strikingly, we found that all ST736 VREfm isolates (n = 111) carried the two common mutations (W73C and T120A) in the *liaFSR* system. Such a high mutation rate in the *liaFSR* system among ST736 strains has not yet been documented in any *E*. *faecium* with other genetic background. Mutations in *liaFSR* have been associated with high daptomycin MICs [16, 20] and failure in treatment of bacteremia caused by daptomycin-susceptible VREfm [52, 53]. Our findings and data from other studies may serve as the basis of a potential diagnostic tool to screen and identify isolates carrying mutations in the *liaFSR* system, and/or isolates with a specific genotype (i.e., ST736) that may predispose to subsequent development of *in vivo* daptomycin resistance, thus to optimize the use of daptomycin against enterococci in clinical settings. Also, it would be worthy to investigate if relatively poor response to daptomycin therapy in patients infected with ST736 strains, as compared to those infected with non-ST736 VREfm strains.

The estimated emergence of ST736 strains in the mid-2000s at our institution seems coincident with the initial clinical use of daptomycin after FDA’s approval in 2003. It is unclear if the co-mutation (W73C and T120A) of the *liaFSR* in ST736 strains resulted from a serial of evolutional events and/or positive selections by daptomycin, and if this genetic alteration indeed contributed to its rapid expansion and dissemination at our institution with a relatively high usage of daptomycin.

Mutations in either *liaFSR* or *cls* alone may not be sufficient in conferring phenotypic resistance to daptomycin in enterococci [21, 45]. In our study, only about one-third (38/111, 34.2%) of ST736 strains carrying the two mutations in *liaFSR* exhibited daptomycin-resistant phenotype (MIC > 4 μg/mL). Also, one VREfm isolate (non-ST736) harboring *cls* mutations was susceptible to daptomycin. Nevertheless, in an *E*. *faecium* clone like ST736 with all strains harboring a predisposing genetic alteration in the *liaFSR*, subsequent mutation in the *cls* gene seems to be sufficient to confer daptomycin resistance. This is based on our observation that all 13 VREfm ST736 isolates with mutations in both *liaFSR* and *cls* exhibit daptomycin-resistant phenotype. It is noteworthy that the most common *cls* mutation among ST736 strains is an asparagine to threonine (N13T) substitution, differing from those (N13I and N13S) reported in enterococci with other genetic background [14, 21, 22, 45]. The N13T substitution of *cls* was previously described as N12T in one California *E*. *faecium* isolate with high-level daptomycin resistance (MIC of >256 μg/mL) [54]. Moreover, the thirteen ST736 isolates carrying mutations in both *liaFSR* and *cls* displayed a daptomycin MIC_50_ of 32 μg/mL (ranging from 8 to >256 μg/mL), which is much higher than that of daptomycin-resistant ST736 isolates without *cls* mutations (MIC_50_ of 8 μg/mL), indicating that co-mutations in both *liaFSR* and *cls* might have predisposed to high daptomycin MICs in ST736 strains. On the other hand, 25 of 38 (65.8%) ST736 DNSEfm strains did not carry *cls* mutations, suggesting diverse mechanisms of resistance to daptomycin in *E*. *faecium* [45] and a necessity of further exploring other alternative genomic pathways associated with daptomycin resistance. Notably, VREfm isolate E253 had a daptomycin MIC of >256 μg/mL. In addition to a mutation in the *cls* (A20D), two new genetic alterations, including mutation in a tyrosine kinase (P90H) and a frameshift in a gene coding the TlyA family rRNA (cytidine-2'-O)-methyltransferase, were also identified on the chromosome of this isolate, as well as mutations in insertion sequence (IS) family transposases on the plasmid. Previously, mutation in histidine kinase has been reported in a daptomycin-resistant *E*. *faecium* isolate [19]. Given the important role of protein kinases in regulating bacterial physiology and stress response [55], it would be interesting to determine if bacterial kinases, including tyrosine kinase described in this study, are associated with daptomycin resistance in enterococci. There was no significant genetic alteration in an isogenic pair of VREfm isolates with different daptomycin MICs (4 μg/mL for E232 and >256 μg/mL for E243), highlighting the complexity and other potential mechanisms such as small RNA [48] and/or differential gene expression that may have involved in daptomycin resistance.

The observed high occurrence of DNSEfm at our institution likely resulted from the unique genetic characteristics of ST736 strains that predispose to daptomycin resistance and nosocomial dissemination of DNSEfm. Previously, we reported that 81.7% (17 of 21) DNSEfm from 2009 to 2012 had a prior exposure to daptomycin [27]. In this study, we noticed that the majority (76.9%, 10 of 13) of DNSEfm isolates with *cls* muations were from patients with prior exposure to daptomycin while hospitalized during 2009 to 2012. The high prevalence of ST736 strains harboring a predisposing mutation in the *liaFRS* system seems to increase the likelihood of developing resistance by either introducing or selecting mutation in the *cls* during daptomycin therapy. Only 6 of 20 (30%) patients with DNSEfm in 2013 had prior exposure to daptomycin. Nosocomial transmission might have occurred and attributed to the observed high prevalence of DNSEfm in 2013 in spite of the implementation of hospital-wide enhanced infection control measures, including the use of ultraviolet environmental disinfection in patient rooms [56].

The limitations of this study include: 1) all VREfm isolates examined were from a single institution; 2) the lack of VREfm isolates between 1996 and 2008 for a more precisely evolutional analysis; and 3) we only examined mutations in *liaFSR* and *cls* that are known to be associated with daptomycin resistance in *E*. *faecium*. Other mutations and additional daptomycin resistance mechanisms are under further investigation.

In conclusion, our study demonstrates an evolutionary change in clonality and antimicrobial susceptibility of *E*. *faecium* population over the past 20 years and a recent emergence of VREfm clone ST736 associated with daptomycin nonsusceptibility at our institution. For clone ST736 strains, resistance to daptomycin likely resulted from predisposing genetic alterations in the *liaFSR*, totaling by mutations in the *cls* and possibly other genes and pathways, through evolution and/or sequential adaption. The high occurrence of ST736 strains with increased risk of developing daptomycin resistance during therapy and nosocomial dissemination of VREfm [57] might have contributed to the observed high prevalence of DNSEfm at our institution.

## Supporting information

S1 File Tables and figure. **Table A. Distribution of clonality among *E*. *faecium* clinical isolates (1995–2013). Table B. Clinical, microbiological and mutation of the *liaFSR* and *cls* genes of daptomycin-nonsusceptible *E*. *faecium* isolates. Table C. Distribution of daptomycin MICs of dominant VREfm clones for each time period of evaluation. Figure A. The whole maximum clade credibility (MCC) tree of ST736 strains.** This is a zoomed-in version of the MCC tree showing the ST736 clade and the estimated divergence time of each node in year.
(PDF)
