# Supplementary material for: Evolution and mutations predisposing to daptomycin resistance in vancomycin-resistant Enterococcus faecium ST736 strains
Source: PLoS One. 2018 Dec 21;13(12):e0209785. doi: 10.1371/journal.pone.0209785 (PMC6303062; doi:10.1371/journal.pone.0209785)
Supplement: S1 File — Table A. Distribution of clonality among E. faecium clinical isolates (1995–2013). Table B. Clinical, microbiological and mutation of the liaFSR and cls genes of daptomycin-nonsusceptible E. faecium isolates. Table C. Distribution of daptomycin MICs of dominant VREfm clones for each time period of evaluation. Figure A. The whole maximum clade credibility (MCC) tree of ST736 strains. This is a zoomed-in version of the MCC tree showing the ST736 clade and the estimated divergence time of each node in year. (PDF) [file pone.0209785.s001.pdf]

Evolution and Mutations Predisposing to Daptomycin Resistance in Vancomycin-Resistant *Enterococcus faecium* ST736 Strains

Guiqing Wang[1, 5, *], Fan Yu[2], Henry Lin[2], Karthikeyan Murugesan[2], Weihua Huang[1], Andrew G. Hoss[2], Abhay Dhand[3], Leslie Y. Lee[4], Jian Zhuge[5], Changhong Yin[1], Marisa Montecalvo[3], Nevenka Dimitrova[2], and John T. Fallon[1, 5]

Supplemental information

Table A. Distribution of clonality among *E. faecium* clinical isolates (1995-2013)[a]

Year	Total no. of isolates	MSLT sequence type (no. of isolates, %)				
		ST17	ST18	ST412	ST736	Miscellaneous[b]
1994-1995	43	33 (76.7)	3 (7.0)	0	0	7 (16.3)
2009	20	0	2 (10.0)	2 (10.0)	9 (45.0)	7 (35.0)
2010	30	0	1 (3.3)	6 (20.0)	16 (53.3)	7 (23.3)
2011	30	0	2 (6.7)	3 (10.0)	23 (76.7)	2 (6.7)
2012	35	0	6 (17.1)	5 (14.3)	20 (57.1)	4 (11.4)
2013	92	0	24 (26.1)	19 (20.7)	43 (46.7)	6 (6.3)
Total	250	33 (13.2)	38 (15.2)	35 (14.0)	111 (44.1)	33 (13.2)

[a] *E. faecium* isolates from 2009 to 2012 are not randomly selected; selective bias may be existed.

[b] Miscellaneous ST for 1994-1995 isolates: ST16 (n = 2), ST20 (1), ST186 (1), ST280 (1) and ST535 (2); for 2009 isolates: ST203 (1), ST282 (4), ST750 (1) and one new ST (1); for 2010 isolates: ST282 (1), ST750 (2), ST893 (1), ST894 (1) and ST896 (1) and ST1314; for 2011 isolates: ST656 (1) and ST896 (1); for 2012 isolates: ST32 (1), ST214 (1), ST584 (1) and ST664 (1); for 2013 isolates: ST117 (2), ST448 (1), ST749 (1), ST895 (1), and ST896 (1). See supplemental table S1 for details.

Table B. Clinical, microbiological and mutation of the *liaFSR* and *cls* genes of daptomycin-nonsusceptible *E. faecium* isolates

E. faecium group	Year	Isolate	Source	MLST sequence type (ST)	Vancomycin MIC (µg/mL)	*van* gene	Daptomycin MIC (µg/mL)	*liaR*.W73C	*liaS*.T120A	*cls*.N13I	*cls*.N13S	*cls*.N13T	*cls*.A20D	*cls*.H215R	*cls*.R218Q	Average depth of Coverage
ST736 (n=38)	2009	E39	Blood	736	>16	*vanA*	96	1	1	0	0	1	0	0	0	460.0
	2010	E13	Blood	736	1	-	32	1	1	0	0	0	0	0	0	86.6
	2010	E14	Blood	736	>16	*vanA*	16	1	1	0	0	0	0	0	0	66.2
	2010	E17	Wound	736	>16	*vanA*	16	1	1	0	0	1	0	0	0	68.2
	2010	E34	Urine	736	>16	*vanA*	48	1	1	0	0	1	0	0	0	21.6
	2010	E53	Wound	736	>16	*vanA*	64	1	1	0	0	1	0	0	0	37.2
	2011	E146	Urine	736	>16	*vanA*	32	1	1	1	0	0	0	0	0	17.4
	2011	E370	Blood	736	>16	*vanA*	16	1	1	0	0	1	0	0	0	59.9
	2011	E371	Blood	736	>16	*vanA*	8	1	1	0	0	0	0	1	0	84.7
	2011	E372	Blood	736	>16	*vanA*	16	1	1	0	0	0	0	1	0	50.3
	2011	E480	Urine	736	1	-	8	1	1	0	0	0	0	0	1	35.8
	2011	E91	Blood	736	>16	*vanA*	64	1	1	0	0	1	0	0	0	24.4
	2012	E165	Urine	736	>16	*vanA*	16	1	1	0	0	0	0	0	0	16.1
	2012	E166	Urine	736	2	-	8	1	1	0	0	0	0	0	0	37.3
	2012	E168	Urine	736	>16	*vanA*	32	1	1	0	0	0	0	0	0	33.3
	2012	E173	Wound	736	>16	*vanA*	32	1	1	0	0	0	0	0	0	74.7
	2012	E390	Wound	736	>16	*vanA*	8	1	1	0	0	0	0	1	0	67.2
	2012	E395	Blood	736	>16	*vanA*	6	1	1	0	0	0	0	0	0	95.0
	2013	E175	Urine	736	>16	*vanA*	8	1	1	0	0	0	0	0	0	15.6
	2013	E203	Blood	736	>16	*vanA*	8	1	1	0	0	0	0	0	0	46.7
	2013	E207	Urine	736	>16	*vanA*	12	1	1	0	0	0	0	0	0	40.8
	2013	E208	Peritoneal fluid	736	>16	*vanA*	6	1	1	0	0	0	0	0	0	21.3
	2013	E218	Urine	736	>16	*vanA*	8	1	1	0	0	0	0	0	0	87.0
	2013	E222	Urine	736	>16	*vanA*	8	1	1	0	0	0	0	0	0	41.3
	2013	E225	Blood	736	>16	*vanA*	6	1	1	0	0	0	0	0	0	67.6
	2013	E226	Peritoneal fluid	736	>16	*vanA*	6	1	1	0	0	0	0	0	0	48.7
	2013	E231	Wound	736	>16	*vanA*	6	1	1	0	0	0	0	0	0	64.9
	2013	E236	SITE	736	>16	*vanA*	6	1	1	0	0	0	0	0	0	86.6
	2013	E237	Blood	736	>16	*vanA*	8	1	1	0	0	0	0	0	0	62.4
	2013	E242	Wound	736	>16	*vanA*	8	1	1	0	0	0	0	0	0	67.8
	2013	E243	Peritoneal fluid	736	>16	*vanA*	>256	1	1	0	0	0	0	0	0	51.8
	2013	E249	Blood	736	>16	*vanA*	8	1	1	0	0	0	0	0	0	103.5
	2013	E251	Urine	736	>16	*vanA*	6	1	1	0	0	0	0	0	0	68.9
	2013	E253	Wound	736	>16	*vanA*	>256	1	1	0	0	0	1	0	0	131.2
	2013	E261	Urine	736	>16	*vanA*	8	1	1	0	0	0	0	0	0	36.1
	2013	E284	Urine	736	>16	*vanA*	6	1	1	0	0	0	0	0	0	14.0
	2013	E347	Blood	736	>16	*vanA*	8	1	1	0	0	0	0	0	0	31.3
	2013	E356	Blood	736	>16	*vanA*	32	1	1	0	1	0	0	0	0	31.7
Non-ST736 (n=15)	2009	E7	Urine	18	1	-	16	0	0	1	0	0	0	0	0	74.1
	2009	E8	Urine	282	>16	*vanA*	16	0	0	0	0	0	0	0	0	30.0
	2009	E49	Blood	750	≤2	-	16	0	0	0	0	0	0	0	1	25.9
	2010	E50	Blood	412	>16	*vanA*	64	0	0	0	0	0	0	0	0	41.3
	2010	E20	Wound	893	2	-	6	1	1	0	0	0	0	0	0	71.7
	2010	E451	Urine	896	>16	*vanA*	6	0	0	0	0	0	0	0	0	47.2
	2011	E376	Blood	656	>16	*vanA*	12	1	1	0	0	0	0	1	0	65.2
	2011	E367	Blood	896	>16	*vanA*	6	0	0	0	0	0	0	0	0	67.0
	2012	E392	Urine	214	1	-	6	0	0	0	0	0	0	0	0	32.0
	2013	E233	Urine	18	>16	*vanA*	6	0	0	0	0	0	0	0	0	39.8
	2013	E245	Urine	18	>16	*vanA*	12	0	0	0	0	0	0	0	0	112.7
	2013	E325	Blood	18	>16	*vanA*	8	1	1	0	0	0	0	0	0	16.8
	2013	E174	Urine	412	>16	*vanA*	12	0	0	0	0	0	0	0	0	28.4
	2013	E302	Peritoneal fluid	412	>16	*vanA*	8	0	0	0	0	0	0	0	0	61.2
	2013	E354	Blood	412	>16	*vanA*	16	1	1	0	0	0	0	0	0	14.3

60.0

Table C. Distribution of daptomycin MICs of dominant VREfm clones for each time period of evaluation

Year	MLST ST	No. of isolates	MIC (µg/mL)								
			≤2	3	4	8	16	32	64	128	≥256
1994-1995	ST17	33	1	1	31						
	ST18	3	2	0	1						
2009-2012	ST18	11	1	1	9						
	ST412	16	5	4	6				1		
	ST736	69	8	8	37	5	5	4	2	1	
2013	ST18	24	4	5	13	2	1				
	ST412	19	5	3	8	1	2				
	ST736	43	5	4	14	16	1	1			2

Figure A

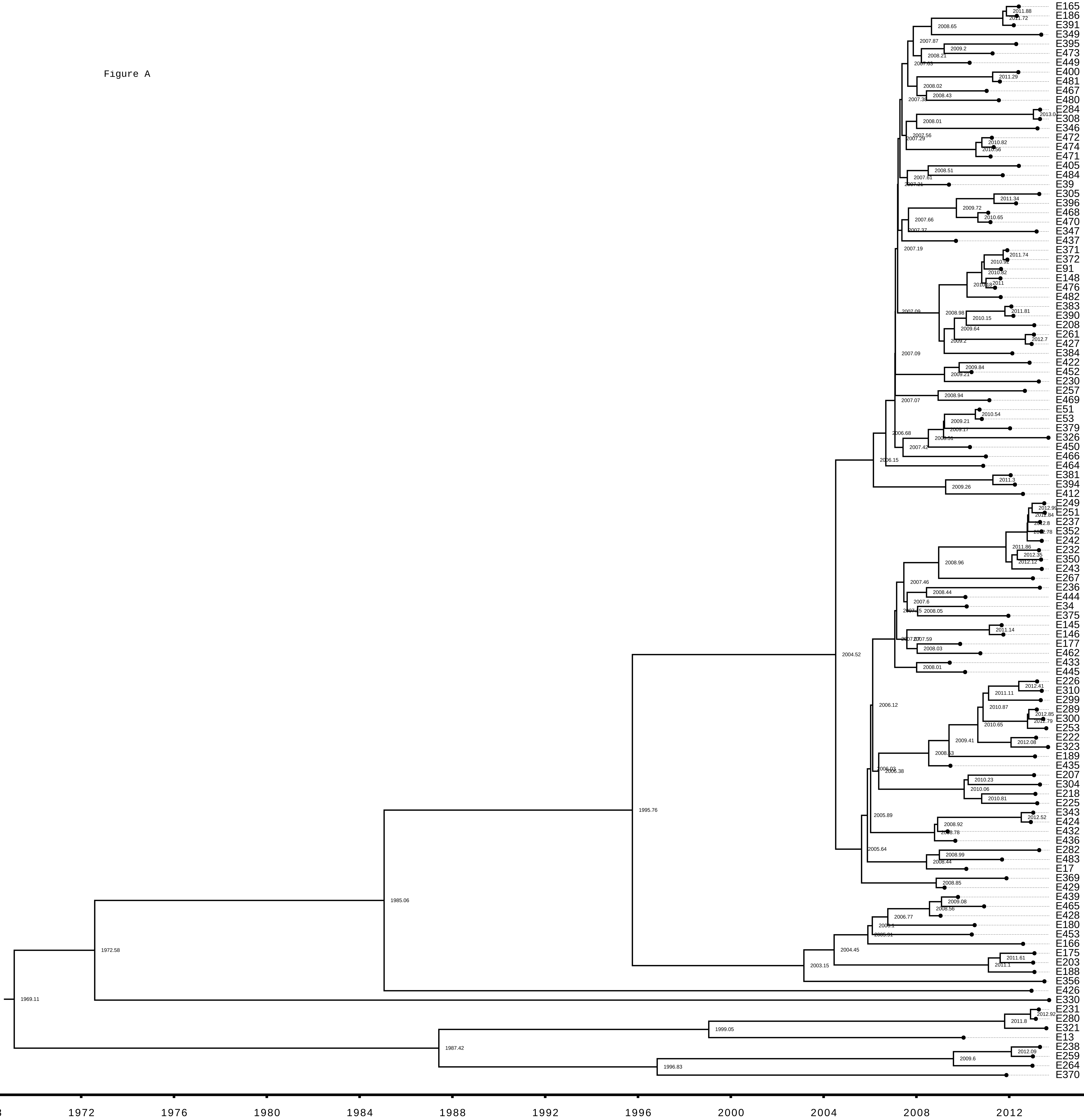

E165
E186
E391
E349
E395
E473
E449
E400
E481
E467
E480
E284
E308
E346
E472
E474
E471
E405
E484
E39
E305
E396
E468
E470
E347
E437
E371
E372
E91
E148
E476
E482
E383
E390
E208
E261
E427
E384
E422
E452
E230
E257
E469
E51
E53
E379
E326
E450
E466
E464
E381
E394
E412
E249
E251
E237
E352
E242
E232
E350
E243
E267
E236
E444
E34
E375
E145
E146
E177
E462
E433
E445
E226
E310
E299
E289
E300
E253
E222
E323
E189
E435
E207
E304
E218
E225
E343
E424
E432
E436
E282
E483
E17
E369
E429
E439
E465
E428
E180
E453
E166
E175
E203
E188
E356
E426
E330
E231
E280
E321
E13
E238
E259
E264
E370
1972
1976
1980
1984
1988
1992
1996
2000
2004
2008
2012
